# Supplementary material for: Blood Immunoproteasome Activity Is Regulated by Sex, Age and in Chronic Inflammatory Diseases: A First Population-Based Study
Source: Cells. 2021 Nov 28;10(12):3336. doi: 10.3390/cells10123336 (PMC8699521; doi:10.3390/cells10123336)
Supplement: Supplementary file 1 [file cells-10-03336-s001.zip › Supplemental Figures.pdf]

Blood Immunoproteasome Activity Is Regulated by Sex, Age and in Chronic Inflammatory Diseases: A First Population-Based Study

Ilona E. Kammerl, Claudia Flexeder, Stefan Karrasch, Barbara Thorand, Margit Heier, Annette Peters, Holger Schulz, Silke Meiners

Supplementary Figures

a)

Workflow	
Part 1	Sample 0.5 ml blood<br>Isolate PBMCs<br>Store cell pellets at -80°C until use<br>↓
Part 2	Isolate native protein (batches of 22, randomized)<br>determine protein concentration<br>Label with ABPs MV151 or LW124/MVB127<br>store labeled lysate at -80°C until use<br>↓
Part 3	Run gel with 4x22 samples in parallel<br>scan gels for ABPs<br>activate protein stain by UV-light exposure<br>scan total protein stain<br>↓
Part 4	densitometry of signals (blinded fashion)<br>quality control and normalization<br>statistical analysis

b)

Quality control		
PBMC isolation	n=924	
Protein isolation	Protein concentration sufficient<br>n=917	Protein concentration too low<br>n=7
Gel scan	Passed quality test<br>n=880 (MV151)<br>n=858 (LW124/MVB127)	Total protein signal insufficient<br>n=44 (MV151)<br>n=66 (LW124/MVB127)

Figure S1. Workflow (A) and quality control steps (**B**) for large-scale analysis of proteasome activities using activity-based probe profiling of peripheral blood mononuclear cells (PBMC) in study participants of the KORA-FF4 study.

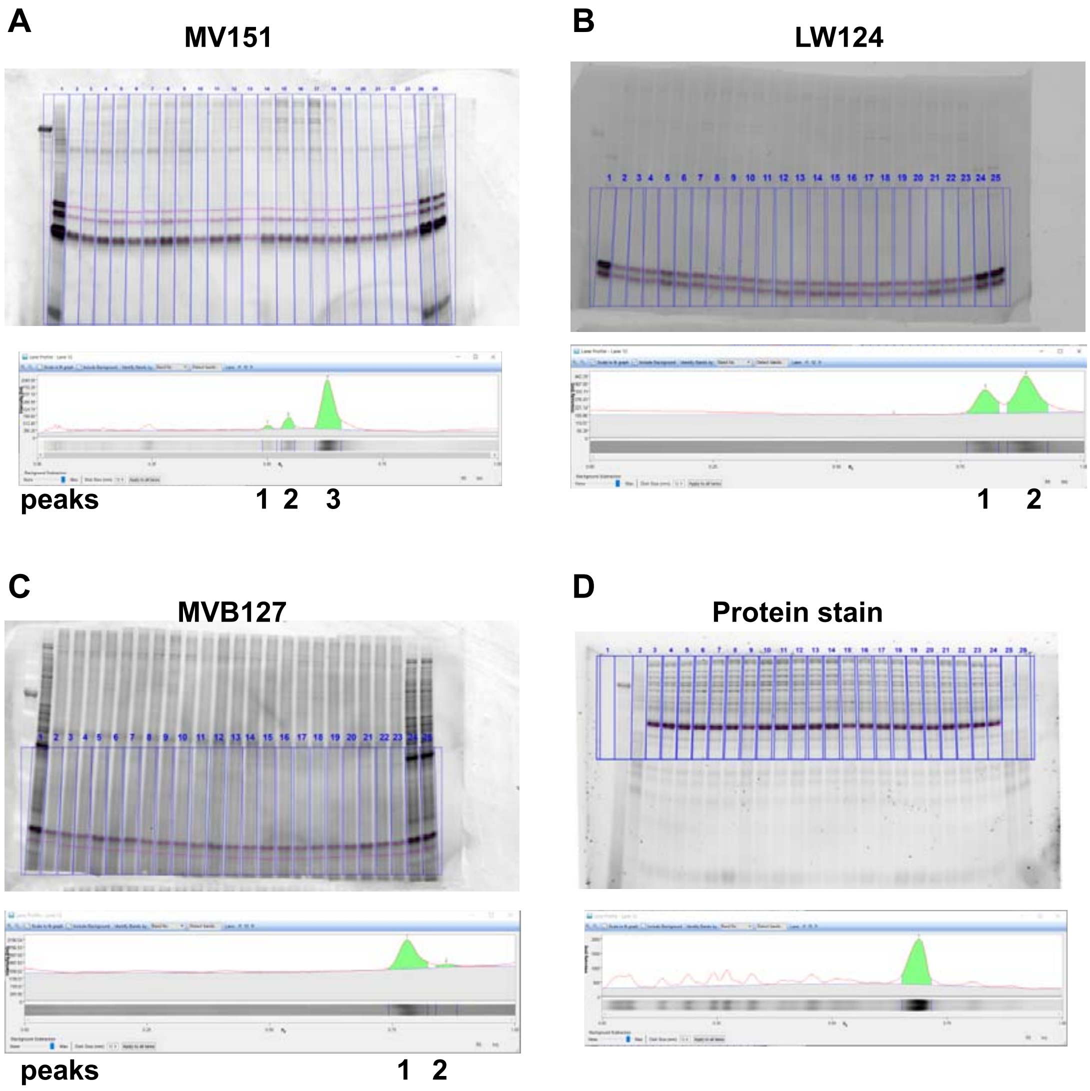

Figure S2: Densitometric analysis of ABPs. Representative images of the different ABP labelings are shown with **(A)** MV151, (**B**) LW124, (**C**) MVB127 and (**D**) protein stain. All gels were loaded as follows: Marker, dummy sample, 22 randomized samples, 2 dummy samples. Dummy samples were loaded to stabilize the gel run and to prevent edge effects. Gels were analyzed with Image Lab software (Bio-Rad). Lane and band detection was performed automatically and adjusted manually if necessary. All lanes had the same width. Background was subtracted automatically and the signal obtained could be assigned to the respective subunits. **(A)** MV151 labeled β2 (peak 1 in the profile of an exemplary lane), MECL1 (peak 2) and the other four β-subunits (peak 3) which cannot be discriminated due to their similar molecular weight. **(B)** LW124 labeled β1 (peak 1 in the lane profile) and LMP2 (peak 2). (**C**) MVB127 labeled LMP7 (peak1) and β5 (peak 2). As the LW124 and MVB127 ABPs differ in their fluorescent label they can be analyzed simultaneously in the same lysates. **(D)** A proprietary compound inside the gels was activated by UV-light to crosslink to proteins and to gain fluorescent properties. For protein loading controls, the very prominent and strongest band at approx. 40 kDa, which was present in every sample, was used. Densitometric analysis was performed as described.

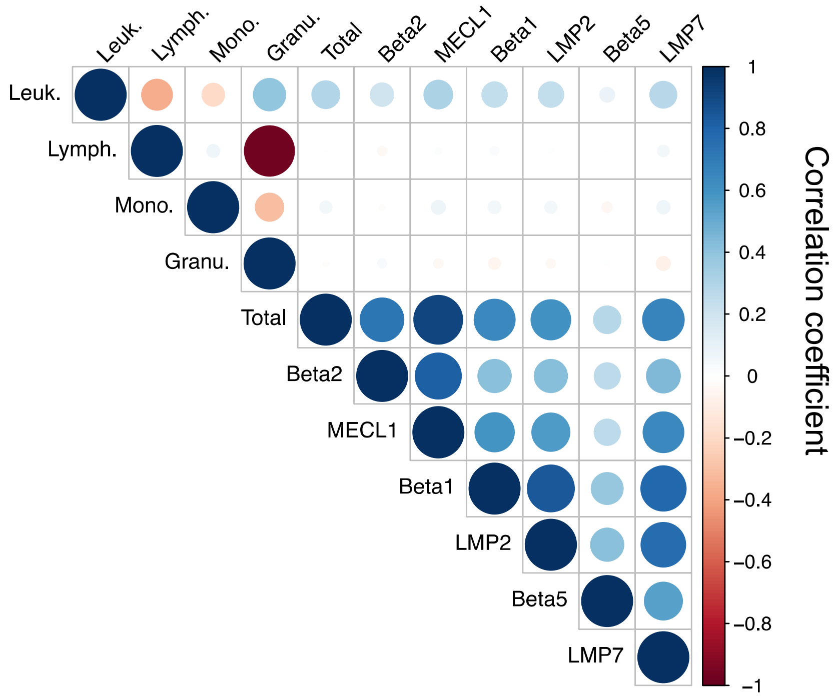

Figure S3: Correlation matrix of proteasome parameters and blood cell populations. Spearman's rank correlation coefficients between standard and immunoproteasome activities with cell counts of leukocytes, percentages of lymphocytes, monocytes and granulocytes are illustrated. Positive correlations are displayed in blue and negative correlations in red color. Color intensity and the size of the circle are proportional to the correlation coefficients.

Males

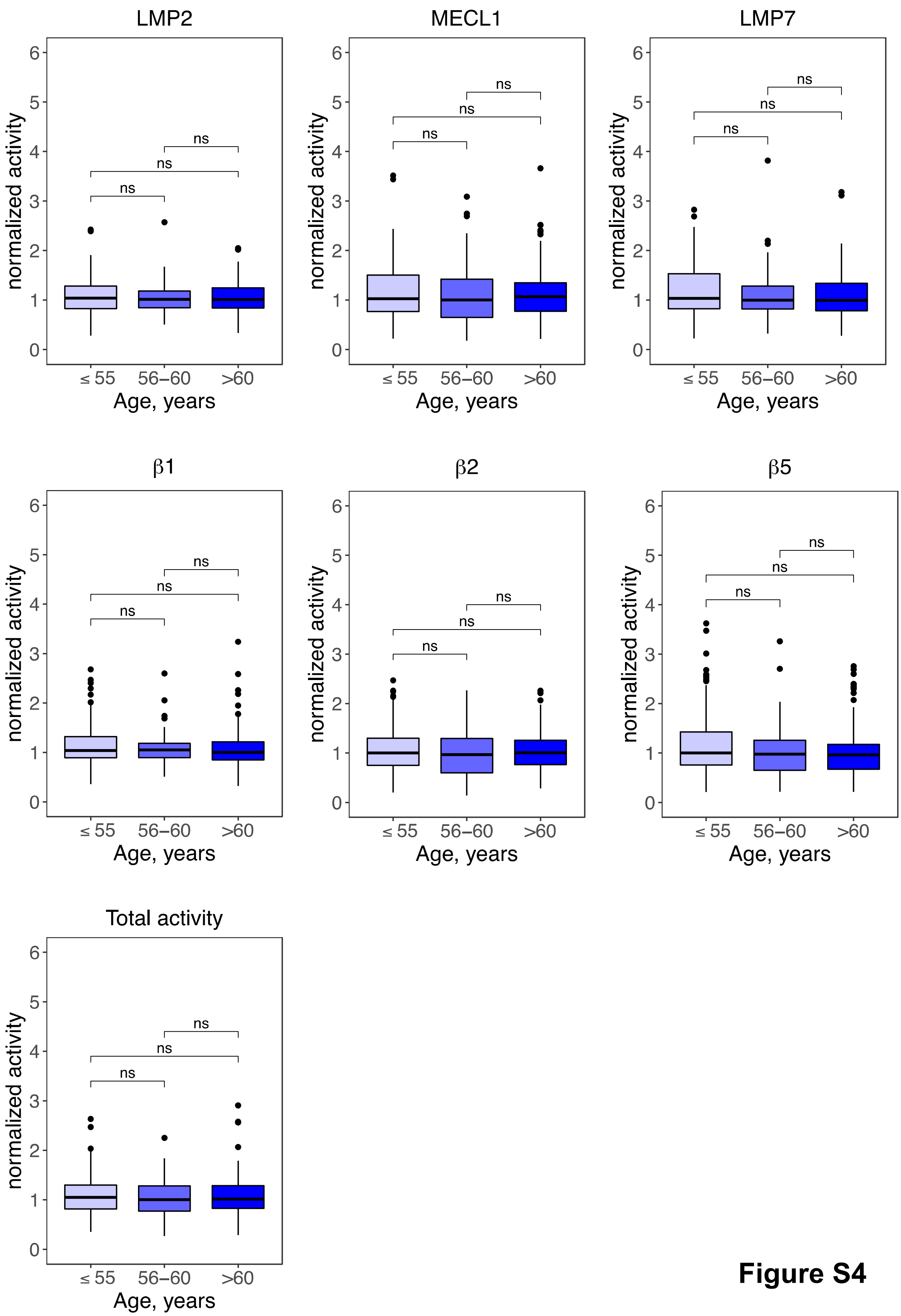

Figure S4

Figure S4: Immunoproteasome activity is not regulated in aging men. Activity-based probe (ABP) analysis of proteasome activity in peripheral blood mononuclear cells (PBMC) of male adults in the KORA-FF4 cohort. All samples were normalized to the batch-specific median. Age was categorized into </= 55, 56-60, and > 60 years of age. Normalized proteasome activities are shown as boxplots of proteasome parameters according to age with median, 1st and 3rd quartile and whiskers indicating +/- 1.5*IQR (interquartile range). Differences between groups were tested using pairwise Wilcoxon rank sum test. ns = non-significant.

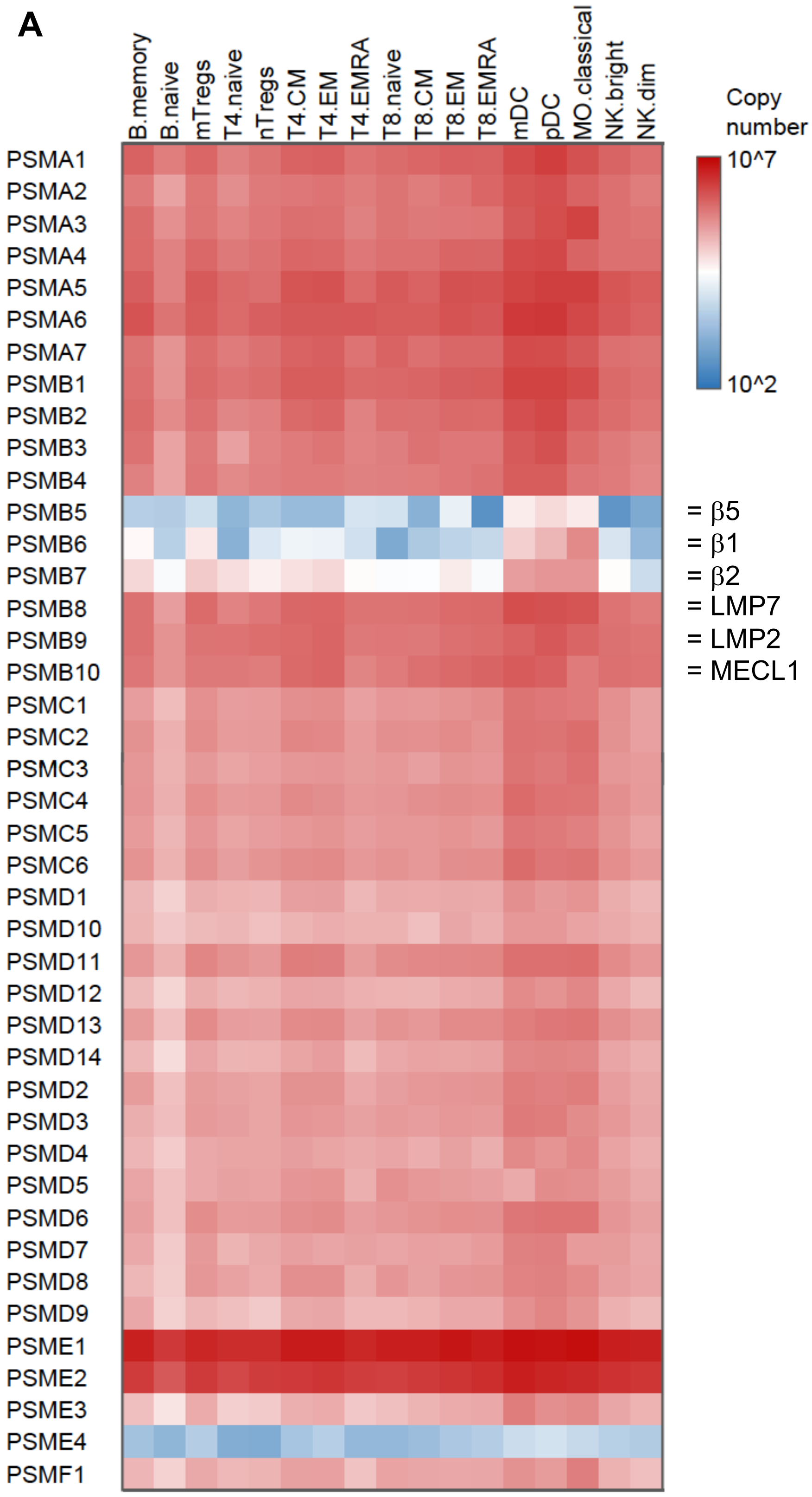

B

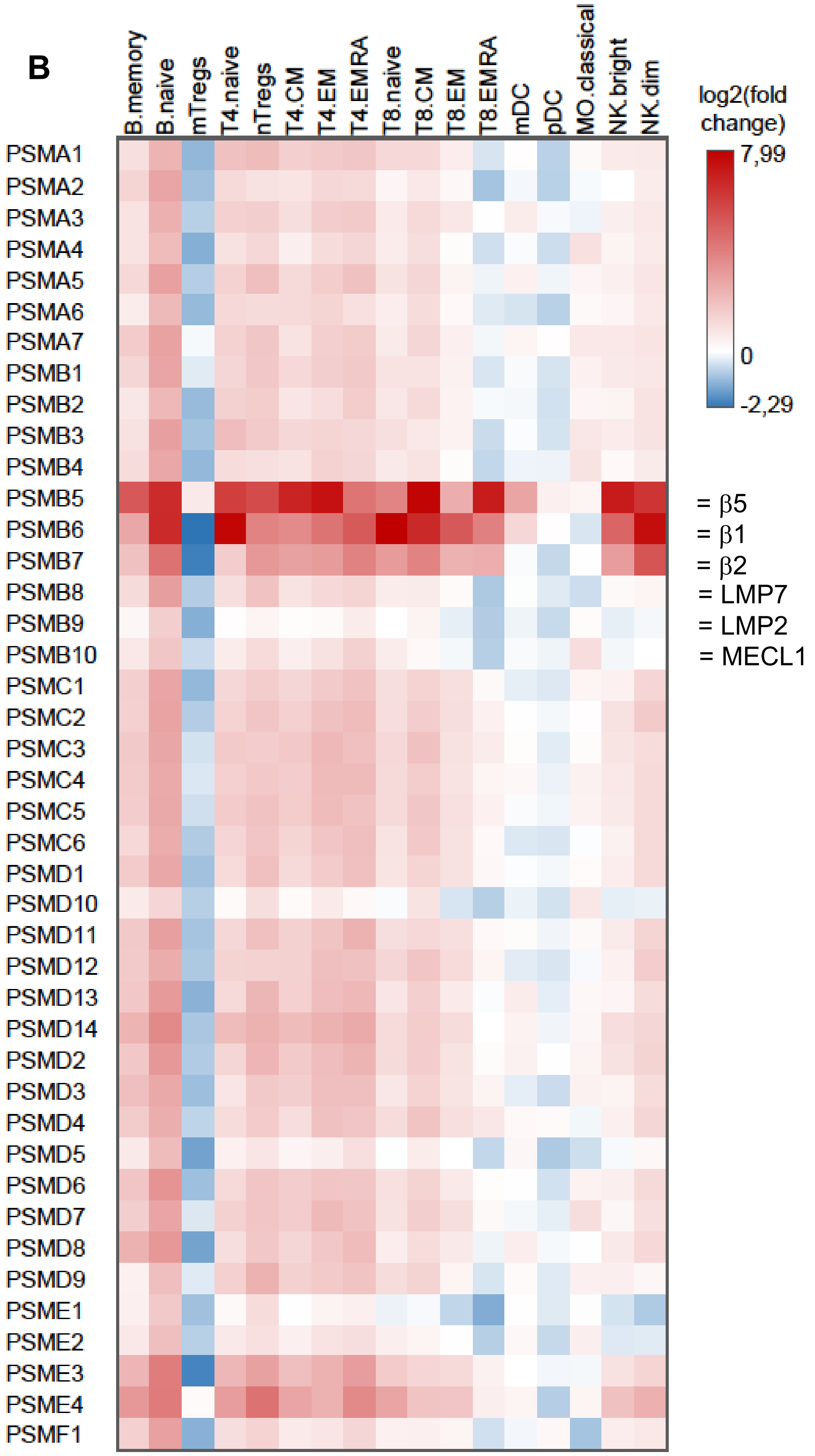

Supplementary Figure S5: Immprot database data on proteasome regulation in human blood-derived immune cells. Data extracted from the public database Immprot[1] where the authors used high-resolution mass spectrometry-based proteomics to characterize 28 primary human hematopoietic cell populations in steady and activated states at a depth of > 10,000 proteins in total. (**A**) Protein copy numbers of the different proteasome subunits (PSMA-F) at steady state. (**B**) Protein copy numbers (given as the relative log2 fold change in activated

primary human hematopoietic cell populations versus steady state situation). Mean values are shown. PSMB5-7; standard proteasome, PSMB8-10: immunoproteasome.

[1] Rieckmann, J. C. *et al.* Social network architecture of human immune cells unveiled by quantitative proteomics. *Nat. Immunol.* 18, 583–593 (2017). http://www.immprot.org/.
